# Ciliopathy-Associated Protein Kinase ICK Requires Its Non-Catalytic Carboxyl-Terminal Domain for Regulation of Ciliogenesis

**DOI:** 10.3390/cells8070677

**Published:** 2019-07-04

**Authors:** Yoon Seon Oh, Eric J. Wang, Casey D. Gailey, David L. Brautigan, Benjamin L. Allen, Zheng Fu

**Affiliations:** 1 Department of Pharmacology, University of Virginia Medical School, Charlottesville, VA 22908, USA; 2 Department of Microbiology, Immunology, and Cancer Biology, University of Virginia Medical School, Charlottesville, VA 22908, USA; 3 Center for Cell Signaling, University of Virginia Medical School, Charlottesville, VA 22908, USA; 4 Department of Cell and Developmental Biology, University of Michigan Medical School, Ann Arbor, MI 48109, USA

**Keywords:** primary cilia, ciliopathy, intestinal cell kinase, ciliopathy-associated protein kinase, ciliogenesis, kinesin family member 3A, and phosphorylation

## Abstract

Loss-of-function mutations in the human *ICK* (intestinal cell kinase) gene cause dysfunctional primary cilia and perinatal lethality which are associated with human ciliopathies. The enzyme that we herein call CAPK (ciliopathy-associated protein kinase) is a serine/threonine protein kinase that has a highly conserved MAPK-like N-terminal catalytic domain and an unstructured C-terminal domain (CTD) whose functions are completely unknown. In this study, we demonstrate that truncation of the CTD impairs the ability of CAPK to interact with and phosphorylate its substrate, kinesin family member 3A (KIF3A). We also find that deletion of the CTD of CAPK compromises both localization to the primary cilium and negative regulation of ciliogenesis. Thus, CAPK substrate recognition, ciliary targeting, and ciliary function depend on the non-catalytic CTD of the protein which is predicted to be intrinsically disordered.

## 1. Introduction

Intestinal cell kinase (ICK) is a highly conserved serine/threonine protein kinase in the CMGC (CDK/MAPK/GSK3/CLK) group of the human kinome [1,2]. ICK was named after the tissue from which it was first cloned, and is now appreciated as a misnomer because ICK is not restricted to the intestine but is instead expressed in a wide variety of tissues. ICK shares significant homology with MAPK in the catalytic domain and contains a MAPK-like TXY motif in the activation loop. However, unlike classic MAPKs it is not acutely activated by growth factors through canonical dual-specificity upstream MEKs [2,3]. Instead, ICK is activated by phosphorylation of Thr157 in its TDY motif by CDK20 (cyclin-dependent protein kinase 20) (also known as CCRK, cell cycle-related kinase) [4]. The tyrosine in this motif undergoes auto-phosphorylation for full activation of ICK [3]. In addition to the TDY motif regulation, ICK may be partially inactivated by fibroblast growth factor (FGF) signaling-mediated phosphorylation of the conserved Tyr15 [5]. 

Multiple studies have demonstrated that ICK and CDK20/CCRK play an evolutionarily conserved role in the assembly and function of primary cilia [6,7,8,9,10,11]. ICK was also identified as a mediator of FGF-induced changes in primary cilia morphology and function [5]. The primary cilium is a microtubule-based organelle that senses and transduces environmental signals to regulate intracellular processes and cell behaviors [12]. Genetic mutations that cause ciliary dysfunction have been identified in human disorders collectively called ciliopathies [13]. Several loss-of-function mutations in the human *ICK* gene have been identified in ciliopathies [14,15,16]. Our mouse model carrying such a ciliopathy mutation (R272Q) in the *ICK* gene died at birth and displayed developmental abnormalities in multiple organ systems, demonstrating that *ICK* is essential for embryonic development [17,18]. Given that the essential role of ICK is in the primary cilium and is associated with ciliopathy, we hereinafter refer to ICK as CAPK, ciliopathy-associated protein kinase.

The molecular mechanisms underlying CAPK signaling and ciliary functions are still largely unknown. In primary cilia, kinesin-2 motor complex (KIF3A/KIF3B/KAP3) mediates anterograde intraflagellar transport (IFT) which is critical for cilium formation and maintenance [19]. KIF3A has been proposed as a direct substrate of CAPK [7]. Here, we demonstrate that CAPK interacts with human KIF3A and phosphorylates a conserved site Thr672 both in vitro and in vivo. We found that the long, unstructured, non-catalytic carboxyl-terminal domain (CTD) of CAPK is required for this interaction with and phosphorylation of KIF3A. We also provide compelling evidence that the CTD of CAPK is essential for not only its ciliary targeting but also its role as a suppressor of ciliogenesis.

## 2. Materials and Methods

### 2.1. Plasmids and Antibodies

pEBG-GST-CAPK plasmids encoding CAPK wild type (WT), kinase dead (KD), and CTD truncation (1–291), as well as pEGFP-CAPK plasmids encoding CAPK WT, KD, R272A, and CTD truncation (1–291) were described in [2,3]. pCIG-HA-KIF3A was described in [20]. 

KIF3A-phospho-Thr672 antibody was generated in rabbits against keyhole limpet hemocyanin-coupled phospho-KIF3A peptide RPR[pT]SKGKARPKTGC at GenScript (Piscataway, NJ, USA). Phosphopeptide-specific antibodies were affinity-purified through a positive selection over phosphopeptide antigens followed by negative selections over non-phosphopeptide antigens. GST-tag (B-14) mouse monoclonal (sc-138) and HA-tag (12CA5) mouse monoclonal (sc-57592) antibodies were from Santa Cruz Biotechnology (Dallas, TX, USA). KIF3A (D7G3) rabbit monoclonal (#8507) and HA-tag (C29F4) rabbit monoclonal (#3724) antibodies were from Cell Signaling Technology (Danvers, MA, USA). Arl13B rabbit polyclonal antibody (17711-1-AP) was from Proteintech (Rosemont, IL, USA). Goat anti-rabbit IgG (Alexa Fluor 594) preadsorbed antibody (ab150084) was from Abcam (Cambridge, MA, USA). 

### 2.2. Cell Culture and Transfection

HEK293T and NIH-3T3 cells were maintained at 37 °C and 5% CO_2_ in Dulbecco’s modified Eagle’s medium (DMEM) supplemented with 4.5 g/L glucose and 10% fetal bovine serum (FBS) or 10% new born calf serum (NBCS). HEK293T cells were transfected using a calcium phosphate protocol as described in [21], and NIH-3T3 cells were transfected using the lipofectamine 2000 reagent following the manufacturer’s instruction.

### 2.3. GST Pull-Down, Immunoprecipitation, and Immunoblotting

Forty eight hours after transfection, cells were lysed in lysis buffer (50 mM Tris-HCl, pH 7.4, 150 mM NaCl, 1% NP-40, 2 mM EGTA, complete protease inhibitors [Roche], 10 mM sodium orthovanadate, 5 mM sodium fluoride, 10 mM sodium pyrophosphate, 10 mM β-glycerophosphate, and 1 µM microcystin LR). Cell lysate was cleared by centrifugation. GST-CAPK proteins were pulled down from cell lysate using glutathione Sepharose 4B beads (GE Healthcare, Chicago, IL, USA) following the manufacturer’s instruction. HA-KIF3A proteins were immunoprecipitated from cell lysate using the HA antibody, and captured on GammaBind Sepharose beads (GE Healthcare). 

Cell extracts or Sepharose beads were boiled for 5 min in an equal volume of 2X Laemmli sample buffer (120 mM Tris-HCl, pH 6.8, 4% SDS, 20% glycerol, 10% β-mercaptoethanol, 0.02% bromophenol blue) and loaded on an SDS gel. Samples were transferred to a PVDF (polyvinylidene difluoride) membrane and blocked for one hour in 5% dry milk before primary antibody incubation in TBS containing 0.1% Tween-20 and 5% bovine serum albumin (BSA) for 90 min at room temperature or overnight at 4 °C. This was followed by extensive rinses and one hour incubation with horseradish peroxidase (HRP)-conjugated secondary antibody. Chemiluminescence signals were developed using Millipore Immobilon ECL reagents (EMD Millipore, Burlington, MA, USA). 

### 2.4. In Vitro and In Vivo Phosphorylation Assay

For in vitro analysis of KIF3A phosphorylation by CAPK, HA-KIF3A was expressed in HEK293T cells, affinity-purified from cell extracts by anti-HA Sepharose beads. HA-KIF3A substrates (0.1–0.5 μg) were incubated with active His-CAPK^1–291^ [4,22] proteins (50 ng) and 100 µM [γ-^32^P]-ATP (PerkinElmer) in kinase buffer [50 mM HEPES (pH 7.5), 10 mM MgCl_2_, 2 mM DTT, and complete protease and phosphatase inhibitor cocktails (Roche)] at 30 °C for 5–30 min. The reaction was quenched by the addition of 2× SDS sample buffer and the samples were processed and analyzed for radioactivity by autoradiograph as described in [4,22]. For non-radioactive assay, the above reaction mixtures were incubated with unlabeled ATP and the reactivity of HA-KIF3A against the KIF3A-phospho-Thr672 antibody was analyzed by Western blot.

For in vivo analysis of KIF3A phosphorylation by CAPK, HA-KIF3A and GST-CAPK were co-expressed in HEK293T cells. HA-KIF3A proteins were immunoprecipitated from cell lysate and captured on anti-HA Sepharose beads. The reactivity of HA-KIF3A against the KIF3A-phospho-Thr672 antibody was analyzed by Western blot.

### 2.5. In Vitro Binding Assay

GST-CAPK was expressed in HEK293T cells and affinity-purified by glutathione Sepharose beads. GST-CAPK beads were incubated with cell lysate containing HA-KIF3A proteins at 4 °C overnight, rinsed extensively, and eluted with 2× SDS sample buffer at 95 °C for 5 min. Eluate was Western blotted against HA or KIF3A antibody for detection of HA-KIF3A proteins bound to GST-CAPK. 

### 2.6. Immunofluorescence Microscopy

NIH-3T3 cells grown on collagen-coated coverslips were fixed by 4% paraformaldehyde (PFA) in PBS, rinsed in PBS, and then permeabilized by 0.2% Triton X-100 in PBS. After one hour in blocking buffer (3% goat serum, 0.2% Triton X-100 in PBS), NIH-3T3 cells on cover slips were incubated with cilia marker Arl13B rabbit antibody at 4 °C overnight followed by rinses in PBS and one hour incubation with anti-rabbit IgG (Alexa Fluor 594-conjugated) secondary antibody. After extensive rinses, slides were mounted in antifade reagent containing DAPI (4′,6-diamidino-2-phenylindole) for imaging (Zeiss AxioImager Z1, Zeiss, Oberkochen, Germany). 

### 2.7. Statistical Analysis

Quantified experimental data were analyzed by the student *t*-test. Data were reported as mean ± standard deviation (SD). *P*-values less than 0.05 were considered as significant.

## 3. Results

### 3.1. CAPK Phosphorylates Human KIF3A-Thr672 In Vitro and In Vivo

**Figure 1:**
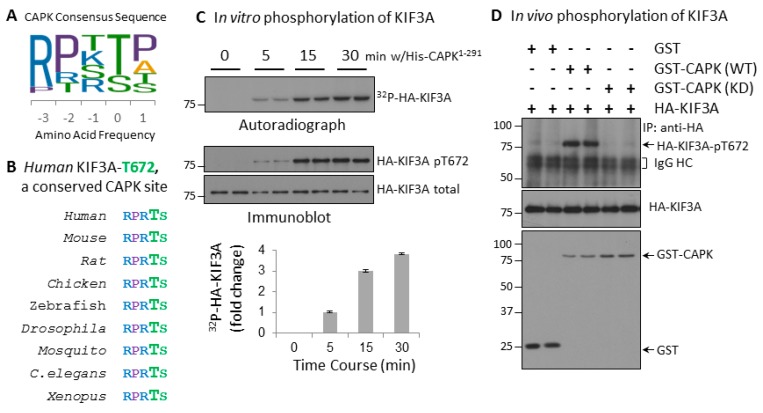
Human KIF3A-Thr672 is a highly conserved CAPK phosphorylation site. (**A**) CAPK has a unique substrate consensus sequence that strongly prefers arginine (R) at −3 and proline (P) at −2 positions. (**B**) Human KIF3A has an evolutionarily conserved CAPK site, Thr672. (**C**) CAPK can phosphorylate KIF3A-Thr672 in vitro. HA-KIF3A were expressed in HEK293T cells, affinity-purified, and then incubated in vitro with active His-CAPK^1–291^ [4]. KIF3A-phospho-Thr672 signals were analyzed by either ^32^P radioisotope labeling shown on autoradiograph or a phosphosite-specific antibody shown on Western blot. (**D**) CAPK can phosphorylate KIF3A-Thr672 in vivo. GST, GST-CAPK WT, or GST-CAPK KD was co-expressed with HA-KIF3A in HEK293T cells. Total and phospho-Thr672-specific signals of HA-KIF3A were assessed by Western blotting of anti-HA immunoprecipitates.

We previously identified the CAPK substrate phosphorylation site consensus sequence R-P-X-pT/S-P/A/T/S as shown in Figure 1A. CAPK shows strong preference for substrates with arginine at −3 position and proline at −2 position, but less stringency for proline at +1 position relative to classic MAP kinases. Human KIF3A-Thr672 is located within a CAPK consensus sequence RPRTS that is conserved among metazoans (Figure 1B) and is phosphorylated by CAPK in vitro [7]. We used both ^32^P radioisotope labeling and a phosphosite specific KIF3A-phospho-Thr672 antibody, to show that KIF3A-Thr672 is phosphorylated by CAPK in vitro (Figure 1C). We further tested whether this phosphorylation occurs in vivo by co-expressing GST alone, GST-CAPK KD or GST-CAPK WT with HA-KIF3A in HEK293T cells. Phosphorylation of KIF3A in anti-HA immunoprecipitates was analyzed by Western blotting with the KIF3A-Thr672 phosphosite specific antibody (Figure 1D). HA-KIF3A-Thr672 was phosphorylated in cells expressing active GST-CAPK, but not in control cells expressing GST or GST-CAPK KD. These data provided evidence that KIF3A-Thr672 is an in vivo target of CAPK.

### 3.2. Deletion of CAPK CTD Compromises Ability to Bind and Phosphorylate KIF3A

**Figure 2:**
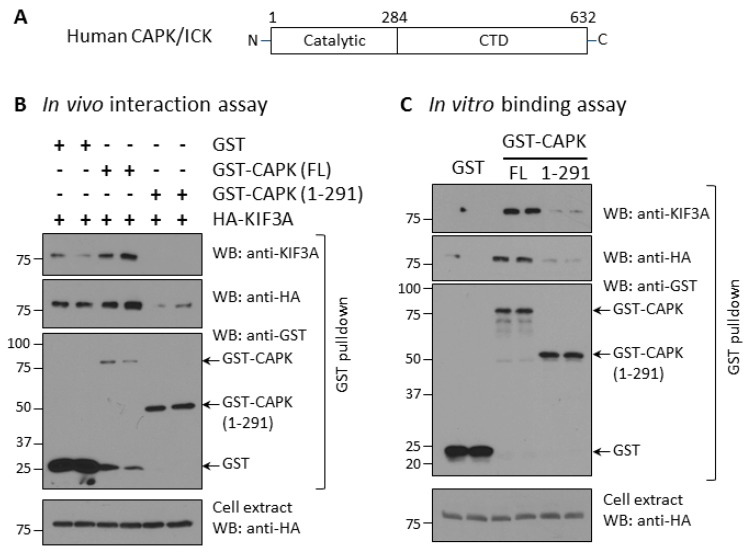
The C-terminal domain (CTD) of CAPK is required for its physical interaction with KIF3A. (**A**) Human CAPK has two basic structural domains: the N-terminal catalytic domain (4–284 aa) and the non-catalytic C-terminal domain (285–632 aa). (**B**) The CTD is required for CAPK interaction with KIF3A in vivo. GST, GST-CAPK full-length (FL), or CTD-truncated (1–291) were co-expressed with HA-KIF3A in HEK293T cells. After GST pull-down, HA-KIF3A bound to GST glutathione beads or in cell extracts were detected by Western blotting against HA or KIF3A antibodies. (**C**) The CTD is required for binding of CAPK to KIF3A in vitro. GST, GST-CAPK FL, or CTD-truncated (1–291) were expressed in HEK293T cells, affinity-purified, and captured on glutathione Sepharose beads. GST beads were then incubated with cell extracts containing HA-KIF3A. HA-KIF3A proteins bound to GST glutathione beads were assessed by Western blotting against HA or KIF3A antibodies.

In contrast to classic MAP kinases, CAPK has a very long (>300 residues) carboxyl-terminal domain (CTD) that shares no significant similarity with known proteins (Figure 2A). We speculated that the CTD may mediate CAPK interaction with other proteins and determine its subcellular localization. We tested whether the CTD of CAPK is required for its interaction with KIF3A. First, we analyzed interaction between CAPK and KIF3A by co-expressing GST, full-length GST-CAPK, or truncated GST-CAPK (1–291) with HA-KIF3A in HEK293T cells (Figure 2B). We used glutathione Sepharose beads to pull down GST fusion proteins from cell extracts and detected HA-KIF3A proteins bound to the beads by anti-HA and anti-KIF3A Western blotting. Full-length GST-CAPK was expressed at low levels, but still effectively pulled down HA-KIF3A. In contrast, the CTD-deleted GST-CAPK (1–291) expressed at higher levels, but pulled down a little of the HA-KIF3A protein that was not detected by anti-KIF3A and barely detected with anti-HA. It is worth pointing out that the GST tag alone, when highly over-expressed as shown on Western blot, non-specifically pulled down some HA-KIF3A proteins, detected by anti-HA immunoblotting (Figure 2B). These data support the conclusion that truncation of the CTD nearly eliminates the interaction between CAPK and KIF3A in living cells.

Furthermore, we conducted an in vitro binding assay to evaluate the interaction between CAPK and KIF3A (Figure 2C). This assay provided better control over the input amounts of the binding partners. We expressed GST, GST-CAPK full-length, or CTD-truncated CAPK in HEK293T cells and purified the proteins on glutathione Sepharose beads. We then incubated these beads with cell extracts containing HA-KIF3A and detected HA-KIF3A bound to the beads as well as total HA-KIF3A in the extracts by Western blot (Figure 2C). The full-length GST-CAPK on beads bound to HA-KIF3A was detected by anti-HA and anti-KIF3A blotting. On the other hand, neither GST alone nor CTD-truncated GST-CAPK (1–291) bound to enough of the input HA-KIF3A to be detected by anti-HA or anti-KIF3A blotting. These results show CAPK binds to KIF3A in vitro and the CTD of CAPK is required for this interaction.

**Figure 3:**
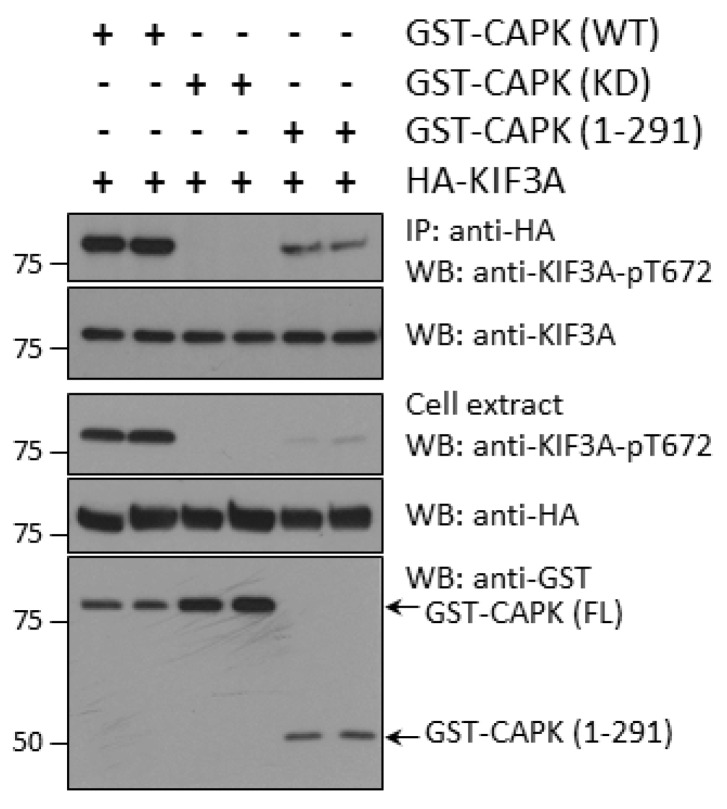
Truncation of the C-terminal domain (CTD) of CAPK compromises its ability to phosphorylate KIF3A-Thr672 in vivo. GST-CAPK wild type (WT), kinase dead (KD), or CTD-truncated (1–291) were co-expressed with HA-KIF3A in HEK293T cells. Phospho-Thr672 signals on HA-KIF3A in anti-HA immunoprecipitates and whole cell extracts were detected by Western blotting with a phosphosite-specific antibody. GST-CAPK proteins were detected with an anti-GST antibody and total HA-KIF3A proteins with both HA and KIF3A antibodies.

Next, we asked the question if the CTD of CAPK influences phosphorylation of KIF3A in vivo. We co-expressed GST-CAPK WT (wild type), KD (kinase dead), or CTD-truncation (1–291) with HA-KIF3A in HEK293T cells. We assessed KIF3A-phospho-Thr672 signals in whole cell extracts and anti-HA immunoprecipitates by Western blotting (Figure 3). Our results show that CTD-truncated GST-CAPK (1–291) produced substantially less phospho-Thr672 in HA-KIF3A as compared with GST-CAPK WT. As a control, GST-CAPK KD showed no phosphorylation of Thr672 in HA-KIF3A. These results suggest that the CTD of CAPK facilitates phosphorylation of Thr672 in KIF3A in vivo.

### 3.3. The CTD of CAPK Is Required for Ciliary Targeting

**Figure 4:**
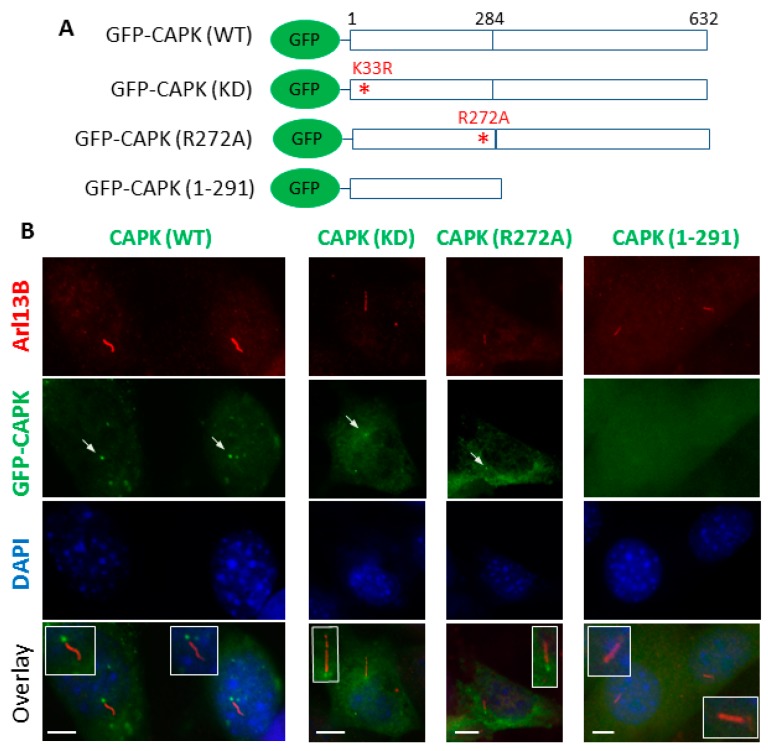
The C-terminal domain (CTD) of CAPK is required for ciliary targeting. (**A**) Schematic diagrams of GFP-CAPK wild type (WT), kinase dead (KD), loss-of-function mutation R272A, or CTD-truncation (1–291) constructs transiently transfected into NIH-3T3 cells. (**B**) NIH-3T3 cells were stained with cilia marker Arl13B antibody followed by Alexa Fluor 594-conjugated secondary antibody (red). Nuclei were stained with DAPI (blue) in the mounting medium. Scale bars, 5 μm.

Mounting evidence has shown localization of CAPK at the primary cilium in cells [7,8,11,15,16,23]. By transiently transfecting GFP-CAPK (Figure 4A) into NIH-3T3 cells, we observed that a fraction of GFP-CAPK was localized at the base of the primary cilium (Figure 4B). Kinase-dead GFP-CAPK mutant proteins (K33R and R272A [3]) were also targeted to primary cilia. This demonstrated that the kinase activity of CAPK was not required for the ciliary localization of the protein. In contrast, CTD-truncated GFP-CAPK (1–291) protein was predominantly diffused in the cytoplasm and not localized to primary cilia (Figure 4B). This novel observation suggests that the CTD of CAPK, and not its kinase activity, is required for ciliary targeting of the kinase.

### 3.4. The CTD Truncation Abolishes the Suppressive Effect of CAPK on Ciliogenesis

**Figure 5:**
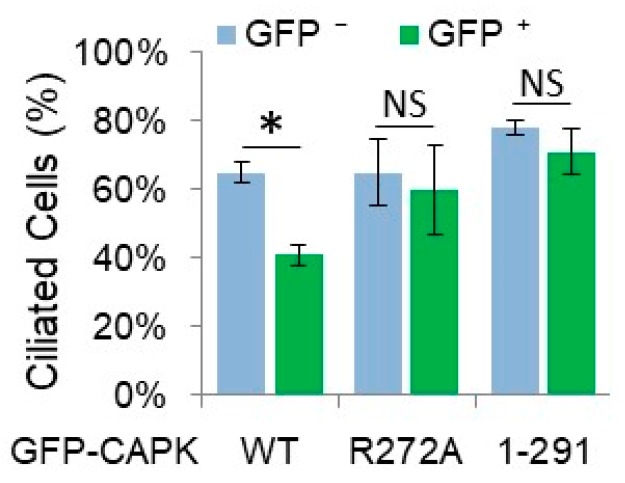
Truncation of the C-terminal domain (CTD) of CAPK impairs its ability to suppress ciliogenesis. GFP-CAPK wild type (WT), loss-of-function mutant (R272A), or CTD-truncated (1–291) proteins were expressed in NIH-3T3 cells. Primary cilia were visualized through immunofluorescence of a ciliary marker, Arl13B. The percentages of ciliated cells in GFP-positive (green bar) and GFP-negative (blue bar) cells were calculated and shown here as mean ± SD, n = 412 cells (WT), 376 cells (R272A), 420 cells (1–291), * *P* < 0.01, NS = not significant.

Since the CTD of CAPK is required for ciliary targeting and binding, and phosphorylation of its ciliary substrate KIF3A, we hypothesized that the CTD is essential for CAPK function in suppressing the formation of primary cilia. We examined ciliogenesis by transfecting NIH-3T3 cells with plasmids encoding GFP-CAPK WT, R272A, or CTD-truncated (1–291) and quantifying ciliated cells in GFP-positive versus GFP-negative cells (as controls) using a ciliary marker, Arl13B. Expression of GFP-CAPK WT reduced the number of ciliated cells by 25%, whereas neither the loss-of-function R272A mutant CAPK protein or the CTD-truncated mutant, GFP-CAPK (1–291) had a significant effect on ciliogenesis (Figure 5). Cells expressing the mutant CAPKs had about the same rate of cilia positive cells as the non-GFP expressing control cells (~60%) (Figure 5). These results demonstrated that the kinase activity and the non-catalytic CTD of CAPK are required for the regulation of ciliogenesis.

## 4. Discussion

Although CAPK is very similar to classic MAPKs in the N-terminal catalytic domain, it diverges significantly in both the length and sequence of the C-terminal non-catalytic domain (CTD). No structural domain or motif has been found in this CTD (285–632 aa) using NCBI’s conserved domain architecture retrieval tool (CDART) [24]. Further, the predicted secondary structure for this CTD is an entirely random coil, with no α-helix or β-strand (Porter 5.0: http://distilldeep.ucd.ie/porter/) [25]. This suggests that the CTD may be another example of an intrinsically disordered protein (IDP) region [26]. Even though IDPs lack stable 3D structures they are known to have functions such as ligand and protein binding [26]. We previously postulated that the CTD of CAPK might be important for determining functional specificity by regulating protein–protein interactions and intracellular localization. Here, we first report two interrelated regulatory roles of the CTD of CAPK: 1) Mediating the physical and functional interaction with its ciliary substrate KIF3A, and 2) regulating CAPK ciliary targeting and suppressive function in ciliogenesis.

A lack of the CTD compromises both ciliary localization and the ability of CAPK to bind and phosphorylate KIF3A. This raises the question of whether cilia localization or phosphorylation of KIF3A is important for the CTD-dependent CAPK effect on ciliogenesis. By comparison R272A is a kinase inactivating mutation [3] that impairs the ability of CAPK to suppress cilia formation, but does not affect its ciliary localization. This observation suggests that localization to the primary cilium is not sufficient for CAPK to negatively regulate ciliogenesis, which favors the hypothesis that phosphorylation of KIF3A is required for the suppressive effect of CAPK on ciliogenesis. We cannot exclude the possibility that CAPK phosphorylation of KIF3A also occurs in an extraciliary location. 

We do provide new compelling evidence supporting human KIF3A-Thr672 (mouse Kif3a-Thr674) as an in vivo phosphorylation site of CAPK. This CAPK site is in the C-terminal region of human KIF3A that contains multiple phosphorylation sites, including PKA site Ser687 and CaMKII sites Thr692 and Ser696 [27]. Phosphorylation of S687/T692/S696 enhances the cargo-binding and trafficking activities of KIF3A [27]. It remains to be determined whether phosphorylation of KIF3A-Thr672 by CAKP is a new mechanism for regulation of KIF3A activity in IFT. A recent elegant study underscored that kinesin-2 motor complex (KIF3A/KIF3B/KAP3)-mediated anterograde IFT is critical for cilium formation [19]. Previously it has been shown that a mouse Kif3a mutant protein containing mutation of all 8 phospho-sites at the C-terminal could not rescue the ciliogenesis defects in Kif3a-knockdown cells [7], consistent with the notion that phosphorylation of KIF3A C-terminal cargo-binding domain is critical for IFT and ciliogenesis. Interestingly, mouse Kif3a-T674A mutant protein exhibited a stronger capacity than Kif3a-WT protein to rescue cilia formation in Kif3a-knockdown cells [7]. This intriguing observation supports our current hypothesis that KIF3A-pThr672 acts as a downstream effector through which CAPK negatively regulates ciliogenesis. Further studies are required to address how CAPK phosphorylation of KIF3A-Thr672 affects IFT and ciliogenesis, and whether deregulation of this phosphorylation event is required for the ciliopathy phenotypes caused by CAPK dysfunction.

## 5. Conclusions

CAPK is a highly conserved modulator of primary cilia structure and function. From this study, we conclude that both the catalytic activity and the intrinsically disordered non-catalytic C-terminal domain (CTD) of CAPK are required for the suppression of ciliogenesis. Here, we propose two mechanisms by which the CTD of CAPK is involved in its ciliary function. Firstly, CAPK localization to the primary cilium depends on the presence of the CTD. Secondly, the CTD facilitates CAPK interaction with and phosphorylation of its ciliary substrate KIF3A.
